# Disease information disclosure among patients with mental illness and their family members in China

**DOI:** 10.3389/fpsyt.2022.1036568

**Published:** 2023-01-04

**Authors:** Yi Wang, Siyao An, Xun Yang, Zhuoqiu Zhang, Shuiying Li, Jing Yao, Ying Chen, Hong Deng

**Affiliations:** ^1^Mental Health Center, West China Hospital, Sichuan University, Chengdu, China; ^2^Sleep Medicine Center, The Third People’s Hospital of Mianyang, Sichuan Mental Health Center, Mianyang, China; ^3^Sichuan Clinical Medical Research Center for Mental Disorders, Chengdu, China; ^4^School of Public Affairs, Chongqing University, Chongqing, China; ^5^Department of Radiology, Huaxi MR Research Center (HMRRC), West China Hospital of Sichuan University, Chengdu, China; ^6^Hope Recovery and Rehabilitation Center, Chengdu, China

**Keywords:** mental illness, disease information, disclosure intention, stigma, family

## Abstract

**Objective:**

The disclosure of mental illness is a first and crucial step in alleviating stigma and promoting mental recovery. However, at present, there is a lack of research on this subject in the Chinese context. Therefore, we conducted this survey among patients with mental illness and their family members and analyzed the influencing factors.

**Methods:**

Questionnaires were distributed to qualified patients with mental illness and their family members, who were enrolled from the inpatient departments of two major mental health centers in China. Hierarchical linear regression analysis was used to evaluate the factors affecting the disclosure of disease information by patients with mental illness and their family members.

**Results:**

A total of 153 patients and 159 family members were included. The percentages of patients and family members who intended to disclose disease information were 34.6 and 18.2%, respectively. Regarding the benefits of being out (BBO), marital status and the number of hospitalizations explained 13.1% of the variance, and stigma explained 4.3% of the variance. Regarding the reasons for staying in (RSI), marital status and family history of mental illness explained 14.4% of the variance, and stigma explained 14.8% of the variance. In the model predicting the influencing factors of family member disclosure, stigma was a predictor of both BBO and RSI, explaining 8.1 and 8.7% of the variance, respectively.

**Conclusion:**

Both patients and their families were more reluctant than willing to disclose. Marital status, number of hospitalizations and family history of mental illness were all influencing factors of patients’ intentions to disclose disease information. Stigma is closely related to disclosure intention and plays an important role in the disclosure intentions of patients and their families. This suggests that the disclosure of disease information is complex, and many factors need to be considered. Disclosure guidelines should be tailored to individuals.

## 1. Background

Mental illness causes 7% of the global burden of disease as measured in DALYs and 19% of all disability years (1). Patients with mental illness often suffer from prolonged illness and impaired social function, which places a heavy burden on their families and society (2). There is a close relationship between the burden of mental illness and stigma. It has been reported that 21.7–49.5% of people with mental illness worldwide believe they are stigmatized (3, 4). Stigma in mental illness is a serious social problem that has multiple consequences for the individuals concerned and their families. Stigma has a negative impact on self-esteem and affects variables associated with recovery from mental illness, including social relationships, adherence to treatment and willingness to seek help (5). Previous studies have explored many methods, such as companion support groups, psychological education or cognitive reconstruction, to reduce stigma and further reduce the burden of mental illness (6).

However, in these methods, the disclosure of disease information is a first and crucial step in alleviating stigma and promoting mental recovery (7). Disclosure refers to the act of seeking attention by disclosing important personal information that may increase the risk of rejection or ostracism. Disclosure involves trust, confidentiality, and authenticity (8). Disclosure is not a one-time event but a process of constant development. This process does not simply involve telling a secret or stating a fact, nor does it always involve achieving inner relief through the disclosure of information (9). There are 4 attributes of disclosure (8): 1. Information of significance to self-concept that is secret, private or unknowable to others. 2. Assistance perceived to be needed to cope with the information. 3. Tolerance for unpredictable results when soliciting help. 4. Divulgence of this information through communication or behavior. 5. Expectation of serious response to the offer of critical information. And from avoiding social contact to spreading information widely, disclosure is divided into five levels (7): 1. Social avoidance. Stay away from crowds and avoid socializing. 2. Secrecy. Go out into the world, but tell no one about their illness. 3. Selective disclosure. Tell people about their illness who seem likely to understand. 4. Indiscriminant disclosure. In the process of getting along with others, when talking about mental illness, patients will not actively conceal their medical history and experience. When disclosing their mental illness, they will also choose to ignore these negative comments. 5. Broadcasting. Patients not only do not conceal their mental illness, but also actively spread their mental illness history and experience, which means that they can spread knowledge about mental illness to people. In the process of spreading information about their mental illness, the individual’s potential goal is to find out those patients who are willing to share their mental history and rehabilitation experience and to help more people navigate mental illness smoothly. This kind of disclosure has cultivated the confidence of patients when they experience mental illness and stigma.

For people with mental illness, disclosure is not all good; it is not black and white, and it has both advantages and disadvantages (7, 10). For example, disclosure can promote individual recovery and promote disease knowledge and social change (10) as well as bring hope to other patients and allow individuals to vent their emotions, deepen their intimate relationships, obtain emotional support, establish mutual trust between partners, obtain better work results, etc. (7, 10–13). However, disclosure can also lead to discrimination, increased stress, increased isolation and divorce (7, 10).

The advantages and disadvantages of disclosure were mainly discussed in the following two relationships: (1) Disclosure of disease information to future employers. Many people with mental illness are unemployed at home. Even in the periods of low unemployment, many people with mental illness cannot find jobs (14). Because employers generally hold negative attitudes toward jobseekers suffering from mental illness, many unemployed persons suffering from mental illness struggle when deciding whether to disclose their illness to potential employers (15). The analysis of unemployed people suffering from mental illness shows that when deciding whether to disclose their mental illness to future employers, those who hold a more cautious attitude will have a higher re- employment rate after 6 months (16). When talking with prospective employers in a private environment, as long as the unemployed with mental illness do not have a strong sense of public stigma, the disclosure of disease information seems to have a positive impact on them (17). (2) Disease information disclosure in intimate relationships. Most patients with mental illness disclose their mental illness to their partners and parents. They are relatively open to family and friends, and less open to acquaintances and colleagues. Patients believed that the support from their partners was the highest and discrimination was the lowest, while support from acquaintances and colleagues was the lowest, and discrimination was the highest (18). It is the most common to disclose disease information to the spouse, and more than 90% of people will tell their close partners about their mental illness. As intimacy decreases, so does openness. Mental illness requires long-term treatment, and receiving treatment may be a sign of the deterioration of mental illness, which may make it more difficult to conceal disease information, thus driving patients to seek professional help and disclose disease information. Those patients who disclose their mental illness to others in intimate relationships seem to get more support than discrimination (19).

Previous studies on information disclosure in mental illness found that 61% of patients with mental illness in the Japanese population were willing to disclose and 39% were unwilling to disclose their mental illness (20). Similarly, among the Dutch population, more people were willing to disclose (approximately 75%) than were not (21). In addition, two-thirds of Canadians with mental illness were willing to disclose, and one-third were not (22). Other research showed that only 39% of patients disclosed their diagnosis in their places of worship in the USA (23) and that 22.58% of the Brazilian population disclosed their diagnosis results (24). Therefore, we suspect that disclosure intentions for mental illness vary across cultures. In other countries of Asian culture, Bril Barniv, S. et al. (10) discussed the influencing factors of disclosure in the context of Israeli culture. But Israel is a West Asian country, which is quite different from the East Asian countries. Additionally, there is still a lack of specialized research on disclosure of mental illness information and the disclosure rate in Korea, North Korea, India, Singapore and other Southeast Asian countries that may be similar to Chinese culture, let alone the disclosure rate. There is still a lack of research on this subject in the Chinese context.

Family members are the main providers of care for people with mental illness. Caring for a person with a mental illness creates social, emotional, behavioral and financial problems for the family, as well as constraints on family members’ personal lives (25). Previous studies have suggested that family members of patients with mental illness also have significant stigma (26, 27). Affected by traditional culture and negative stereotypes, they avoid the outside world, such as by refusing to let patients go to school or work and refusing help from relevant public welfare organizations, resulting in the social withdrawal of patients and family self-isolation (28, 29). Therefore, family members play a very important role in the process of mental illness, affecting the treatment and rehabilitation of patients with mental illness (30, 31). Therefore, we jointly studied family members’ disclosure intentions and the influencing factors to further understand the differences and connections between family members and patients.

A series of tutorials describe the definition of disclosure and its advantages and disadvantages, under what circumstances disclosure should be made, to whom disclosure should be made, and in what way disclosure should be made, etc. The discloser needs to learn constantly in order to develop the best skills and strategies. There are many layers to this process (32). Therefore, we preliminarily discussed disclosure intention and its influencing factors among mental illness patients and their families in the Chinese context and laid a foundation for future in-depth research. We made the following assumptions: 1. In the context of Chinese culture, patients and their families tend not to disclose information about mental illness. 2. Disclosure intention is highly correlated with stigma.

## 2. Materials and methods

### 2.1. Study participants

Chinese residents who had previously been diagnosed with a mental illness (intellectual disability was excluded) or were family members of a person with a mental illness could be included in the study. A total of 153 patients and 159 family members of patients with mental illness were included. The included patients were from the inpatient departments of the Mental Health Center of West China Hospital of Sichuan University and the psychiatric department of the Third People’s Hospital of Mianyang (Sichuan Mental Health Center). This study was approved by the Ethics Review Committee of Sichuan University and the Third People’s Hospital of Mianyang. Before completing the survey, all patients and their families were informed of the purpose and content of the study.

### 2.2. Procedure

Between September 2020 and September 2021, the hospitals’ electronic medical record system (His system) was used to check medical records and screen inpatients in the mental health center of West China Hospital of Sichuan University and the psychiatric department of the Third People’s Hospital of Mianyang. Then, researchers went to the bedside to explain the purpose, content and general process of the study to eligible patients and their families (if patients are accompanied by their families during hospitalization), to obtain the informed consent. If eligible patients were hospitalized alone, we asked if they could provide their family members’ contact information, so as to obtain their family members’ informed consent to fill in the questionnaire. Then, the patients version questionnaire was distributed to eligible patients, and the family version questionnaire was distributed to eligible family members. As patients or their family members filled in the questionnaire, the researchers were available to answer their questions. The questionnaire was presented through the Questionnaire Star software. Patients or family members could answer questions directly by clicking on links. After each answer was completed and submitted, the questionnaire software recorded the specific data. We set up the questionnaire to be submitted after each question had been answered. Therefore, the returned questionnaires had no missing values. Finally, the completed questionnaires were collected, and the data were analyzed.

### 2.3. Measures

The Consumer and Family Decision Making Scale (CFDMS) (33) was used to assess the opinions of patients on their daily autonomous decision-making. Chen Ying et al. constructed the scale specifically for the Chinese population. The scale consists of a total of 27 items across the following 4 factors: mental care and treatment, personal and social function, community and daily living, and fund management (the Cronbach’s alpha coefficients were 0.86, 0.89, 0.87, and 0.76, and the retest reliability values were 0.81, 0.89, 0.80 and 0.88, respectively). Each item is rated on a scale of 1 to 10 to indicate the percentage of patients who made decisions, for an overall score ranging from 27 to 270, with higher scores indicating greater autonomy in decision-making. With an established content validity, acceptable test-retest reliability, meaningful factorial structure, and good internal consistency, the CFDMS appears to be an acceptable instrument to measure consumers’ and family members’ views of daily decision making. Both patients and their families completed this scale.

Self-stigma was assessed with the Self-stigma of Mental Illness-Scale-Short Form (SSMIS-SF) (34). The scale was translated by the (Chengdu, Sichuan, China) with the permission of Professor Patrick Corrigan from the Illinois Institute of Technology. Although the initial 40-item version of the SSMIS was shown to have strong reliability and validity, the testing time was too long and contained offensive items that prevented the completion of the test (35, 36). The simplified version with 20 items deleted was adopted in this study; this version has been proven to be a reliable and effective tool, and the Chinese version has been proven to have good internal consistency and retest reliability (37). The simplified version shortens the test time and omits offensive questions (34, 38). It consists of four subscales. Each subscale has five items, each of which is scored on a scale from 1 to 9 (1 = strongly disagree, 9 = strongly agree). The total score of the scale is determined by adding the five items contained in each of the four subscales, with the total score ranging from 20 to 180 (34). The higher the score is, the higher the degree of stigma and the stronger the stigma (37). Both patients and their families completed this scale.

Mental health recovery was measured with the Recovery Assessment Scale-Revised (RAS-R). An exploratory and confirmatory analysis was conducted on 24 items out of 41 items in the original scale, and a total of 5 factors were obtained: personal confidence and hope, willingness to seek help, goal and success orientation, dependence on others, and independence from symptoms. The Cronbach’s alpha coefficients of the five factors ranged from 0.74 to 0.87, indicating that 24 items of RAS (RAS-24) are sufficient to assess recovery (39–41). The Chinese version of the RAS-24 (RAS-C) has also been proven to have good internal consistency and psychometric characteristics (42). The scale adopts a 5-point Likert scale (1 = strongly disagree, 5 = strongly agree). The total score is the sum of all items (between 24 and 120 points), and the higher the score is, the better the recovery (43, 44). This scale only needed to be completed by the patients.

The General Self-Efficacy Scale (GSES) was used to assess an individual’s positive self-belief in coping with life’s needs. It was first proposed by Jerusalem and Schwarzer in Germany in 1979 (45). The original 20-item scale was reduced to 10 items in 1981, and the scale has since been translated into 33 languages, including Chinese, and widely used. The Chinese version of the GSES has previously been shown to have high sensitivity and validity. The Cronbach’s alpha for internal consistency for the use of the GSES was 0.805 (46). The scale uses a 4-point Likert scale to calculate the total score, with 1 point indicating completely incorrect and 4 points indicating completely correct. The total score ranges from 10 to 40 points, and the higher the score is, the stronger the sense of self-efficacy (47). This scale only needed to be completed by the patients.

The Coming Out with Mental Illness Scale (COMIS) was translated by the Hope Recovery and Rehabilitation Center with the permission of Professor Patrick Corrigan, Illinois Institute of Technology, USA. This scale was originally developed by Patrick Corrigan et al. (48) in 2010 and revealed two structures through exploratory factor analysis: the benefits of being out (BBO) and the reasons for staying in (RSI). Because the scale was being used for the first time in China, we also calculated its reliability and validity: the Cronbach’s alpha of BBO and RSI were 0.922 and 0.940, and the KMO values were 0.910 and 0.936, respectively. The values were all greater than 0.7, and the *p* values were all less than 0.001, indicating that the scale has good reliability and validity. The scale consists of 21 items, each of which has a 7-point agreement scale (1 = strongly disagree, 7 = strongly agree). Items 1–7 represent BBO. The total score is the sum of the seven items. The higher the score is, the more benefits of disclosure. Items 8–21 represent RSI, and the total score is the sum of 14 items. The higher the score is, the more reasons for not disclosing. Both patients and their families completed this scale.

### 2.4. Statistical analysis

The Chinese version of SPSS 25.0 (IBM Company, Chicago, IL, USA) was used to perform data analysis. The predictors of BBO and RSI were assessed through hierarchical linear regression analysis with the forced entry of three blocks of covariates: demographics as the first block, the patients’s level of recovery as the second block, and degree of stigma as the third block. All the data are suitable for regression analysis.

## 3. Results

Among the patients included in the study, there were 65 males and 88 females, and the mean age, mean years of education, mean age of onset, and mean number of hospitalizations were 26.56, 13.65, 21.61, and 2.22, respectively. Among them, 27.5% had a positive family history of mental illness. Approximately 71.2% had never been married, 9.2% lived alone, 39.9% were currently unemployed, and 34.6% intended to disclose their mental illness. The mean scores of the CFDMS, RAS-R, GSES and SSMIS-SF were 179.10, 87.10, 24.24 and 82.65, respectively. Meanwhile, the average BBO and RSI scores of the COMIS were 25.37 and 66.35, respectively. In addition, 34.6% of the family members of the included mental patients were male, with an average age of 45.99 and years of education of 11.66. Among them, 93.7% had been married before, and 18.2% intended to disclose patients’ mental illness. The average CFDMS and SSMIS-SF scores were 176.05 and 81.12, respectively. The BBO and RSI scores of the COMIS were 21.31 and 57.82, respectively (Table 1).

**TABLE 1:** Description of the sample population.

Variable	Patients	Family members
Male gender	65 (42.5%)	55 (34.6%)
Age	26.56 ± 9.489	45.99 ± 10.537
Years of education	13.65 ± 3.534	11.66 ± 3.987
Age at first onset	21.61 ± 7.852	
Number of hospitalizations	2.22 ± 2.777	
Positive family history	42 (27.5%)	
Marital status		
Ever been married	44 (28.8%)	149 (93.7%)
Never married	109 (71.2%)	10 (6.3%)
Living situation		
Living alone	14 (9.2%)	
Live with others	139 (90.8%)	
Professional status		
Currently working	92 (60.1%)	
No job at present	61 (39.9%)	
Intend to disclose	53 (34.6%)	29 (18.2%)
CFDMS	179.10 ± 45.310	176.05 ± 47.538
RAS-R	87.100 ± 16.920	
GSES	24.24 ± 7.947	
SSMIS-SF	82.65 ± 28.353	81.12 ± 30.408
COMIS		
BBO	25.37 ± 12.447	21.31 ± 12.454
RSI	66.35 ± 22.218	57.82 ± 24.078

### 3.1. Predictors of disclosure intention of patients with mental illness and their families

Table 2 shows the results of hierarchical linear regression analysis of patients with mental illness. In model 1a, marital status and the number of hospitalizations were predictors of willingness to disclose, explaining 13.1% of the variance. Marital status and family history of mental illness were predictors of reluctance to disclose, explaining 14.4% of the variance. In model 1b, the rehabilitation level of patients was not a predictor of whether patients disclosed, but marital status and the number of hospitalizations were still predictors of willingness to disclose. In model 1c, the degree of stigma significantly predicted BBO and explained 4.3% of the variance, and the number of hospitalizations was still a predictor of willingness to disclose. For RSI, stigma explained 14.8% of the variance, and marital status was still a predictor (Table 2).

**TABLE 2:** Predictors of BBO and RSI for patients.

Model statistics	Entered variables	BBO				RSI			
		*B*	S.E.	Beta	*P*	*B*	S.E.	Beta	*P*
Model 1a BBO *F*_9,152_ = 2.403, *p* = 0.014, *R*^2^ = 0.131 Model 1a RSI *F*_9,152_ = 2.671, *p* = 0.007, *R*^2^ = 0.144	Gender	-3.514	2.066	-0.140	0.091	5.455	3.661	0.122	0.138
	Age	-0.284	0.203	-0.217	0.164	-0.181	0.360	-0.077	0.616
	Years of education	-0.334	0.292	-0.095	0.256	0.005	0.518	0.001	0.992
	Marital status	-6.276	2.980	-0.229	0.037	-14.012	5.281	-0.286	0.009
	Family history	-3.664	2.220	-0.132	0.101	-8.167	3.933	-0.165	0.040
	Living situation	5.458	3.624	0.127	0.134	10.405	6.422	0.135	0.107
	Professional status	-3.104	2.061	-0.122	0.134	-1.673	3.652	-0.037	0.648
	Age at first onset	-0.034	0.227	-0.021	0.881	0.029	0.403	0.010	0.942
	Number of hospitalizations	1.032	0.369	0.230	0.006	1.242	0.654	0.155	0.060
Model 1b BBO *F*_12,152_ = 3.026, *p* = 0.006, *R*^2^ = 0.206 Model 1b RSI *F*_12,152_ = 2.624, *p* = 0.083, *R*^2^ = 0.184	Gender	-3.634	2.001	-0.145	0.072	4.923	3.622	0.110	0.176
	Age	-0.363	0.198	-0.277	0.069	-0.122	0.359	-0.052	0.735
	Years of education	-0.309	0.285	-0.088	0.280	0.047	0.516	0.007	0.928
	Marital status	-6.332	2.880	-0.231	0.030	-14.202	5.213	-0.290	0.007
	Family history	-4.002	2.171	-0.144	0.067	-6.882	3.930	-0.139	0.082
	Living situation	5.730	3.523	0.133	0.106	11.803	6.377	0.154	0.066
	Professional status	-2.727	2.027	-0.108	0.181	-2.183	3.668	-0.048	0.553
	Age at first onset	0.013	0.221	0.008	0.953	-0.079	0.401	-0.028	0.844
	Number of hospitalizations	1.039	0.395	0.232	0.010	0.542	0.716	0.068	0.450
	CFDMS	0.032	0.023	0.118	0.154	0.084	0.041	0.171	0.042
	RAS-R	0.065	0.088	0.088	0.464	-0.302	0.160	-0.230	0.062
	GSES	0.226	0.179	0.144	0.210	0.292	0.324	0.104	0.370
Model 1c BBO *F*_13,152_ = 3.535, *p* = 0.006, *R*^2^ = 0.248 Model 1c RSI *F*_13,152_ = 5.308, *p* < 0.001, *R*^2^ = 0.332	Gender	-3.150	1.962	-0.125	0.111	6.538	3.302	0.146	0.050
	Age	-0.320	0.194	-0.244	0.101	0.022	0.327	0.009	0.947
	Years of education	-0.283	0.278	-0.080	0.311	0.132	0.469	0.021	0.778
	Marital status	-5.556	2.825	-0.203	0.051	-11.618	4.756	-0.237	0.016
	Family history	-4.035	2.120	-0.145	0.059	-6.990	3.569	-0.141	0.052
	Living situation	4.614	3.463	0.107	0.185	8.083	5.829	0.105	0.168
	Professional status	-3.002	1.981	-0.118	0.132	-3.101	3.335	-0.069	0.354
	Age of first onset	0.012	0.216	0.008	0.954	-0.081	0.364	-0.029	0.824
	Number of hospitalizations	1.080	0.386	0.241	0.006	0.677	0.650	0.085	0.300
	CFDMS	0.018	0.023	0.066	0.425	0.036	0.038	0.074	0.344
	RAS-R	0.114	0.088	0.155	0.198	-0.138	0.148	-0.105	0.353
	GSES	0.208	0.175	0.133	0.236	0.234	0.295	0.084	0.428
	SSMIS-SF	0.096	0.034	0.220	0.006	0.322	0.058	0.410	0.000

Table 3 shows the results of hierarchical linear regression analysis of family members. From model 2a and model 2b, it can be concluded that the general demographic characteristics and the decision-making ability of patients were not predictors of disclosure. In model 2c, stigma was a predictor of both BBO and RSI, explaining 8.1 and 8.7% of the variance, respectively (Table 3).

**TABLE 3:** Predictors of BBO and RSI for family members.

Model statistics	Entered variables	BBO				RSI			
		*B*	S.E.	Beta	*P*	*B*	S.E.	Beta	*P*
Model 2a BBO *F*_4,158_ = 1.542, *p* = 0.193, *R*^2^ = 0.039 Model 2a RSI *F*_4,158_ = 1.624, *p* = 0.171, *R*^2^ = 0.040	Gender	-0.876	2.095	-0.034	0.677	-2.576	4.046	-0.051	0.525
	Age	-0.049	0.109	-0.041	0.656	0.017	0.211	0.007	0.937
	Years of education	-0.622	0.254	-0.199	0.015	-0.083	0.490	-0.014	0.866
	Marital status	1.550	4.715	0.030	0.743	-19.114	9.106	-0.193	0.037
Model 2b BBO *F*_5,158_ = 1.516, *p* = 0.239, *R*^2^ = 0.047 Model 2b RSI *F*_5,158_ = 0.315, *p* = 0.733, *R*^2^ = 0.041	Gender	-1.124	2.103	-0.043	0.594	-2.437	4.078	-0.048	0.551
	Age	-0.052	0.109	-0.044	0.634	0.019	0.212	0.008	0.930
	Years of education	-0.645	0.254	-0.206	0.012	-0.070	0.493	-0.012	0.887
	Marital status	1.797	4.714	0.035	0.704	-19.252	9.142	-0.195	0.037
	CFDMS	0.025	0.021	0.094	0.239	-0.014	0.040	-0.027	0.733
Model 2c BBO *F*_6,158_ = 3.727, *p* < 0.001, *R*^2^ = 0.128 Model 2c RSI *F*_6,158_ = 3.732, *p* < 0.001, *R*^2^ = 0.128	Gender	-0.698	2.021	-0.027	0.730	-1.584	3.907	-0.031	0.686
	Age	-0.063	0.105	-0.053	0.549	-0.003	0.203	-0.001	0.987
	Years of education	-0.469	0.248	-0.150	0.061	0.282	0.480	0.047	0.557
	Marital status	1.071	4.528	0.021	0.813	-20.708	8.753	-0.209	0.019
	CFDMS	0.013	0.020	0.051	0.509	-0.036	0.039	-0.072	0.354
	SSMIS-SF	0.120	0.032	0.293	0.000	0.241	0.062	0.304	0.000

## 4. Discussion

In this study, we investigated the disclosure intentions of patients with mental illness and their families and analyzed the factors influencing their disclosure and non-disclosure. Among the participants, 34.6% of the patients with mental illness were willing to disclose their illness, while 18.2% of the family members of patients with mental illness were willing to disclose information about the patients’ mental illness. This also confirms hypothesis 1. In addition, this study also found that marital status, number of hospitalizations, family history of mental illness and stigma all played different roles in the disclosure intentions of patients with mental illness. For the family members of patients, among the variables studied, only stigma affected their disclosure intentions.

Among the included patients with mental illness, 34.6% were willing to disclose their illness, which is lower than the percentages of patients with mental illness willing to disclose their illness in Japan, Netherlands and Canada mentioned in the background section (61, 75 and 68%, respectively). This may be because mental illness is not as easily accepted by the public as hypertension and diabetes in Chinese culture. Due to the slow social and economic development in the early years and the late start of psychiatric medicine, patients suffering from mental illness often cannot receive timely and effective diagnosis and treatment (49). The damage to the brain and individual social function caused by mental diseases is very serious. Patients with mental illnesses who cannot be diagnosed and treated in time and effectively often appear in the public view as having “dangerous, uncontrollable, abnormal behavior,” which even affects the social image of mental illness and perceptions of public safety (50–52, 53). A study found that 67.6% of participants agreed that society discriminates against people with mental disorders more seriously than other disabled people. In this study, only 31.40% of the residents held a positive attitude toward psychiatric illnesses (54). Psychiatry in China has developed rapidly in recent years, and while a minority of the public have a certain degree of understanding of mental illness, the majority of the general public still have a negative and stereotyped understanding of mental illness conveyed by the media (52, 54, 55). Therefore, disclosing one’s mental illness is equivalent to telling the general public, “I am potentially at risk.” Against such a backdrop, more people naturally choose not to disclose this information publicly.

Among the family members of patients with mental illness, 18.2% were willing to disclose information about patients with mental illness. Previous studies showed that 48% of the families of patients in India were willing to disclose (56) and that between 59 and 69% of family caregivers in Hong Kong and Beijing endorsed hiding mental illness (57). Like that of patients, in the context of Chinese culture, the willingness to disclose of families of patients with mental illness is lower than that in other countries. In addition to the above stereotype of mental illness, which has an impact on the attitude toward disclosure, another possible reason is that “face” is a social construction deeply rooted in Chinese culture and represents the social status of a person or a family (58, 59). Patients with mental illness may have a negative impact on the social status of their families in the community, resulting in the loss of “face” for their families.

In addition, this study also analyzed the factors affecting the disclosure of patients and their families. We found that marital status, number of hospitalizations, family history of mental illness and stigma all played different roles in the disclosure intention of patients with mental illness. Compared with people with mental illness who had never been married, people who had been married identified both more benefits of disclosure and more reasons to be unwilling to disclose. This is not consistent with previous studies. Carolyn S. Dewa et al. found that those who did not disclose were more likely to be single/never married than those who did (60). A possible reason was that partnership is a very important part of interpersonal relationships. People who have experienced marriage may have a deeper understanding and experience of their partners’ attitudes toward mental illness. Regarding a family history of mental illness, those with a positive family history were more reluctant to disclose than those with a negative family history. A possible reason is that for those with a positive family history, another identity of the patients is also a family member. The patients may look at the problem from the perspective of the disclosure implementer/recipient and have a more comprehensive personal experience of the negative impact of mental illness on the sick person and his or her family. Regarding the number of hospitalizations, the more hospitalizations there were, the more benefits of disclosure that were identified. A reason may be that for those with mental illness at work, frequent hospitalizations lead to more time off from work, and disclosure can allow them to make some adjustments in the workplace (61).

Notably, this study found that for both patients and family members, the stronger their sense of shame, the more benefits of disclosure they identified, and the more reasons they identified for being unwilling to disclose. This is consistent with previous studies; that is, there is a close relationship between stigma and disclosure intention (39, 62, 63), which confirms hypothesis 2. The possible reasons are as follows: on the one hand, a sense of shame has many negative effects for patients (62) and their families (56, 64, 65), which makes them more reluctant to disclose. On the other hand, compared with people with less stigma, people who have stronger stigma will receive more benefits if they choose to disclose because disclosure is a first and crucial step to reduce stigma. However, the predictor of stigma was less explanatory for BBO than RSI (patients: 4.3 < 14.8%; family members: 8.1 < 8.7%). A possible reason is that whether they are influenced by internal or public stigma, people who hesitate to disclose instinctively choose not to disclose, and they subconsciously occupy a dominant position. However, people need to think repeatedly before they can realize the benefits of disclosure so that the proportion of stigma in considering the benefits of disclosure is low.

## 5. Limitations

First, there were small sample sizes of both patients and their families, and fewer teenagers and elderly patients were included than patients of other ages. Second, each model contained too many predictors. Third, most of the included people came from the Sichuan and Chongqing areas in China, and there was a lack of people from more economically developed coastal areas and relatively underdeveloped areas. Fourth, when creating the questionnaire, the extent, object and manner of disclosure were not taken into account, so there were differences in the disclosure scenarios considered by the subjects when filling in the questionnaire. Fifth, there was no further division of the categories of family members and the length of care. Sixth, there were more inpatients (relatively severe symptoms) and fewer outpatients (relatively mild symptoms).

## 6. Conclusion

Our study explored the intentions of psychiatric patients and their families to disclose psychiatric information and analyzed the influencing factors of disclosure. The study found that both patients and their families were more reluctant than willing to disclose this information. Marital status, number of hospitalizations and family history of mental illness were all influencing factors of patients’ intentions to disclose disease information. Stigma is closely related to disclosure intention and plays an important role in the disclosure intentions of patients and their families. This suggests that the disclosure of disease information is complex, and many factors need to be considered, including individual circumstances.

## Data availability statement

The raw data supporting the conclusions of this article will be made available by the authors, without undue reservation.

## Ethics statement

The studies involving human participants were reviewed and approved by Ethics Committee of West China Hospital of Sichuan University, Ethics Committee of the Third People’s Hospital of Mianyang. Written informed consent to participate in this study was provided by the participants’ legal guardian/next of kin.

## Author contributions

All authors listed have made a substantial, direct, and intellectual contribution to the work, and approved it for publication.
